# Coronary ostial angioplasty for juvenile Takayasu arteritis involving the coronary artery using external iliac artery grafts

**DOI:** 10.1007/s11748-022-01838-y

**Published:** 2022-06-12

**Authors:** Tomohiro Iwakura, Shuichiro Takanashi, Akio Masuda, Miyu Hayashida, Go Haraguchi, Mamoru Nanasato, Mitsuaki Isobe, Tomoki Shimokawa

**Affiliations:** 1 Department of Cardiovascular Surgery, Sakakibara Heart Institute, 3-16-1, Asahi-cho, Fuchu-shi, Tokyo, 183-0003 Japan; 2 Department of Cardiology, Sakakibara Heart Institute, 3-16-1 Asahi-cho, Fuchu-shi, Tokyo, 183-0003 Japan

**Keywords:** Takayasu arteritis, Coronary artery ostial lesion, Coronary ostial angioplasty, External iliac artery

## Abstract

Takayasu arteritis can affect the coronary ostia, leading to myocardial ischemia. Coronary ostial angioplasty effectively treats coronary artery ostial lesions associated with Takayasu arteritis. We present a case of juvenile Takayasu arteritis with bilateral subclavian artery occlusions treated with a novel coronary artery ostial angioplasty using the external iliac artery.

**Supplementary Information:**

The online version contains supplementary material available at 10.1007/s11748-022-01838-y.

## Introduction

Takayasu arteritis (TAK) is often associated with coronary artery ostial lesions [1] and treated using corticosteroids and immunosuppressive agents. However, refractory cases are treated with infliximab or tocilizumab (TCZ) [2]. Treating severe coronary artery stenosis is challenging with medication alone and often requires surgery [3], such as coronary artery bypass grafting (CABG), percutaneous coronary intervention (PCI), and ostial coronary artery angioplasty. However, long-term restenosis rates are higher after PCI. Herein, we report a case of successful coronary artery ostial angioplasty using an external iliac artery (EIA) graft.

## Case report

### Medical history

A 15-year-old girl with TAK complaining of chest pain at rest was referred to our hospital. She was diagnosed with TAK according to the 1990 American college of rheumatology classification and modified Ishikawa’s criteria [4] and administered prednisolone (10 mg/day), methotrexate (14 mg/week), and TCZ (162 mg/week) for 6 months. She had a history of Sjögren’s syndrome and was receiving oral prednisone 5 mg/day since 6 years before TAK diagnosis.

### Investigations

**Fig. 1:**
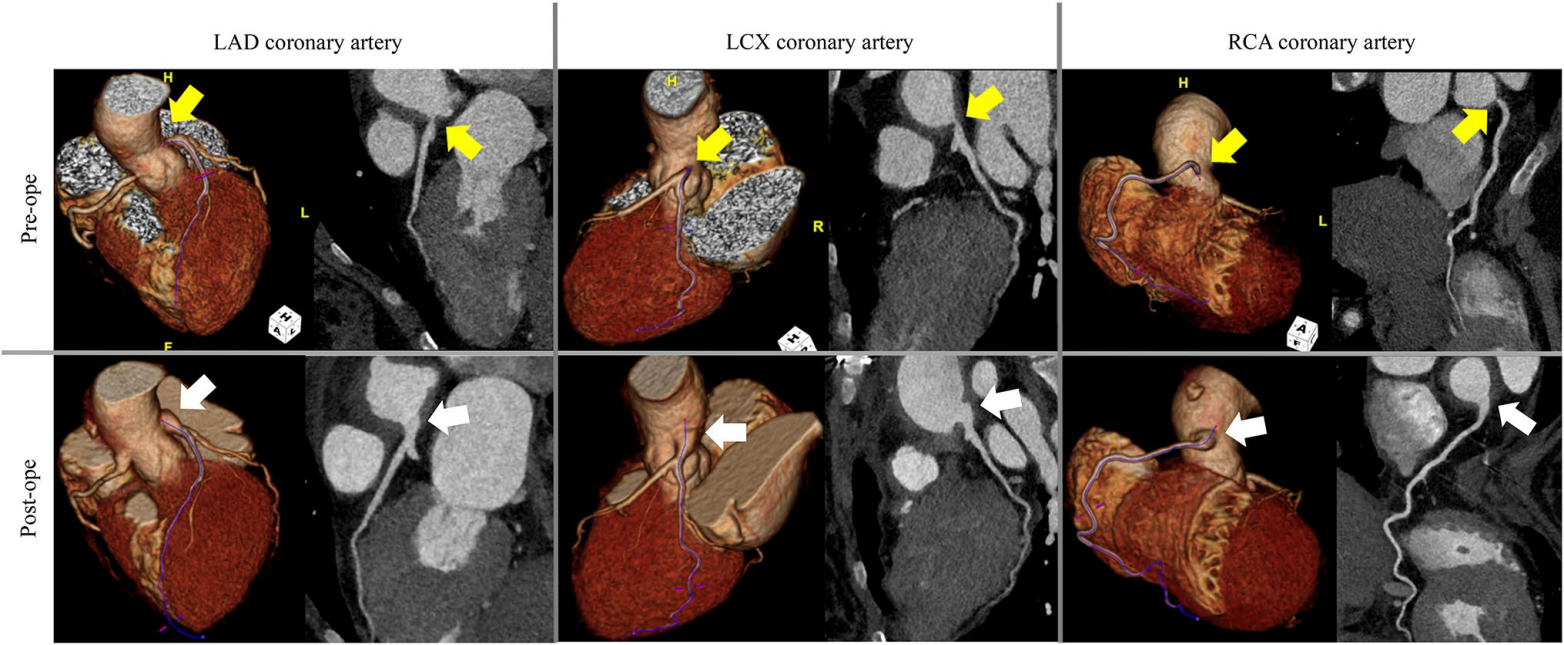
Preoperative and postoperative multiplanar reconstruction computed tomography images of each coronary artery. Yellow arrows indicate the coronary ostial lesion. White arrows indicate enlargement of the left main coronary artery ostium using the external iliac artery patch. *LAD* left anterior descending artery, *LCX* left circumflex artery, *Pre-ope*, preoperative, *Post-ope* postoperative, *RCA* right coronary artery, *EIA* external iliac artery

**Fig. 2:**
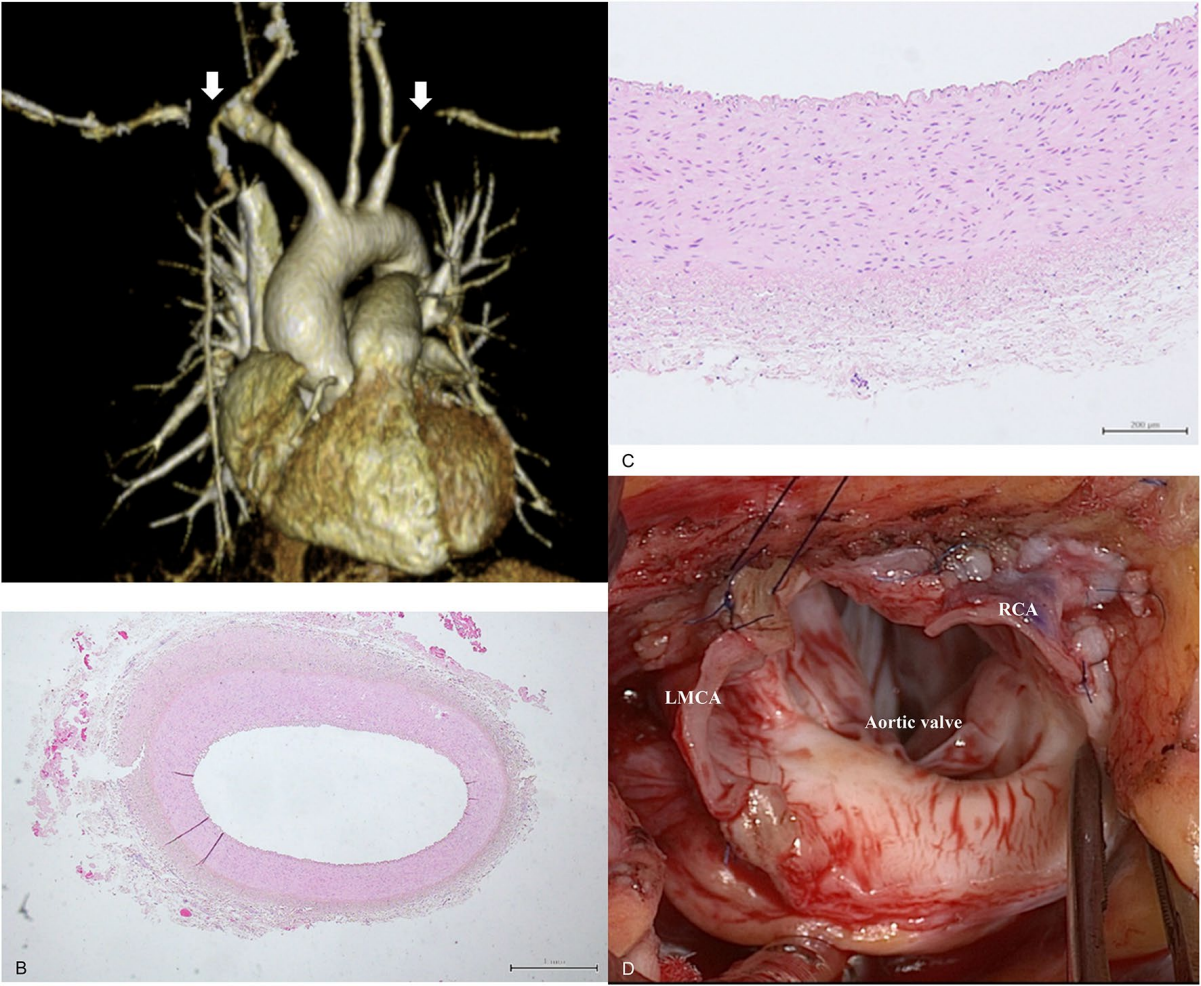
**A** Preoperative three-dimensional computed tomography. White arrows indicate bilateral subclavian artery occlusion. **B**,** C** Histological appearance of the external iliac artery patch specimens demonstrating a muscular artery with no abnormalities in the tunica intima, media, or adventitia and well-preserved internal and external elastic plates, **D** Intraoperative findings and suturing of the external iliac artery patch to enlarge the coronary artery ostium

Coronary computed tomography (CT) revealed 75% right coronary artery (RCA) ostial stenosis and 90% left main coronary artery (LMCA) ostial stenosis (Fig. 1). Contrast-enhanced CT revealed an ascending aortic diameter of 31 mm, right and left subclavian artery occlusion (Fig. 2A), and bilateral carotid artery stenosis extending to the descending and abdominal aorta. IL-6 signaling mediated by binding to its receptor was inhibited during TCZ administration. In addition, C-reactive protein and other inflammatory biomarkers were negative. However, positron emission tomography (PET)-CT showed increased fludeoxyglucose accumulation in the aortic wall (Online resource 1). We suspected an active lesion due to arteritis. Echocardiography showed no wall motion abnormality or decreased ejection fraction. However, ammonia N13 myocardial blood flow PET showed extensive ischemia (Online resource 2).

Six months later, TCZ initiation did not result in regression of the coronary lesions, and surgical intervention was planned. Coronary artery bypass surgery for lesions due to TAK is indicated as an emergency evacuation procedure because the said lesions may regress. Moreover, the bypass may be occluded by future immunosuppressive treatment. In the present case, this procedure was performed for a young patient with TAK who did not have bilateral internal thoracic arteries, and lesions confined to the coronary artery ostium could be expected to be the outcome of a definitive surgery.

### Management

A 40-mm left lateral oblique incision was made for retroperitoneal harvesting of 8 × 60 mm of the left EIA. The excised artery was replaced with a vascular prosthesis (Triplex advance, 8 mm; Terumo, Tokyo, Japan). The lower extremity ischemia time for collecting the EIA graft was 12 min. EIA was histologically muscular throughout its length (Fig. 2B, C). The aorta was transected 15 mm above the left and right coronary artery origins. Epiaortic echocardiography showed aortic circumferential wall thickening (5–7 mm) from the aortic root to distal aortic arch. The aortic root was thoroughly dissected approximately 15 mm proximal to the coronary artery to fully expose RCA and LMCA; this revealed a stenotic orifice, observed through the aortic lumen. The tunica intima was stretched due to aortitis, and the fibroproliferative tunica media and adventitia protruded into the coronary artery ostia. Severe intimal thickening narrowed the RCA orifice.

Coronary artery ostial angioplasty was performed as described by Arai et al. [5, 6]. First, the aortic wall incision was made perpendicular to the right coronary artery and further extended approximately 8 mm into the right coronary artery to a lesion-free area. The EIA graft was incised longitudinally to create a rectangular patch. The patch was anastomosed from RCA to the aortic wall using a 7-0 monofilament suture at the distal end of the RCA incision (Online resource 3). The incised coronary artery was sutured to ensure that its length and the circumference of the EIA patch were roughly the same. Additionally, the aortic wall incision line and long axis incision line of the EIA patch were anastomosed to the same length as the EIA circumference. Second, the LMCA was incised through the aortic wall (Online resource 4). An EIA graft was used for reconstruction (Fig. 2D).

Antiplatelet therapy with aspirin (100 mg/day) was initiated. Prednisone (10 mg/day) and methotrexate (14 mg/day) were continued. TCZ was discontinued for 2 months. CT angiography performed 10 days postoperatively revealed sufficiently dilated and normal reconstructed coronary artery ostia (Fig. 1). The patient was discharged on postoperative day 14.

### Follow-up

99 mTc drug loading scintigraphy performed 2 months postoperatively revealed decreased Tc accumulation in the basal septum, with an inverse redistribution at rest; the decrease was more pronounced at rest. The patient reported overall recovery 3 months later, and chest pain did not recur. No complications were reported.

## Discussion

Coronary ostium angioplasty is useful for coronary artery ostial lesions due to TAK. TAK causes granulomatous inflammation in the ascending aorta, leading to significant proliferation and thickening of the fibrous intima and tunica media of the ascending aortic wall [7]. Coronary artery ostial lesions are often caused by these intimal thickenings protruding into the lumen of the coronary artery to form stenotic lesions. Therefore, replacing the coronary artery from the ascending aorta with a continuous patch may resolve coronary artery ostial stenosis and prevent recurrence [8]. Coronary artery stenosis may improve after immunosuppressive treatment. Therefore, this surgical procedure is theoretically more reasonable than CABG or PCI if the lesion is confined to the coronary artery ostium [9].

Arterial grafts, great saphenous vein, and autologous pericardium are the best patch materials for coronary angioplasty. However, arterial grafts may be histologically superior to great saphenous vein and autologous pericardium due to their anti-inflammatory, antithrombotic, and anti-restenosis properties [10]. Although the internal thoracic artery (ITA) is considered the best for routine CABG, it is not used for TAK because it is often underdeveloped due to subclavian artery stenosis or occlusion and its elasticity, possibly being a source of major lesions in patients with TAK. In this patient, the bilateral subclavian arteries were occluded. Therefore, ITA could not be used as a bypass graft or patch material.

EIA is a muscular artery, and a graft of sufficient anatomical length and diameter can be harvested with minimal risk. The superficial and common femoral arteries are muscular arteries that are usually not involved in arteritis; thus, they can serve as good patch grafts. However, harvesting a sufficient length and diameter is difficult. Additionally, with the harvesting of the superficial and common femoral arteries, the approach site for future testing and treatment can be lost. Furthermore, EIA is easier to handle during anastomosis than the femoral artery, great saphenous vein, and autologous pericardium due to the equal thickness and suppleness of the arterial wall. This procedure is unlikely to result in restenosis of the coronary artery ostium, even if active aortitis further persists. This is precise because more than half of the aortic wall connected with the coronary artery is replaced using EIA, which is less susceptible to aortitis reoccurrence. Thus, based on our findings, EIA can be considered the best patch material for coronary ostial angioplasty in patients with TAK. Although there are no similar reports, our short-term results were good.

## Conclusion

Patch angioplasty could be an excellent alternative for stenotic coronary ostium repair in patients with TAK. However, long-term outcomes must be evaluated.

## Supplementary Information

Below is the link to the electronic supplementary material.

Online Resource 1. PET-CT shows increased FDG accumulation in the aortic wall (white arrows). (DOCX 384 KB)

Online Resource 2. N13 ammonia myocardial blood flow PET shows extensive ischemia. (DOCX 167 KB)

Online Resource 3. RCA angioplasty: intraoperative video. (MPG 61144 KB)

Online Resource 4. LMCA angioplasty: intraoperative video. (MPG 62028 KB)
