# Supplementary figures and images for: Coronary ostial angioplasty for juvenile Takayasu arteritis involving the coronary artery using external iliac artery grafts
Source: Gen Thorac Cardiovasc Surg. 2022 Jun 12;70(12):1005–8. doi: 10.1007/s11748-022-01838-y (PMC9663377; doi:10.1007/s11748-022-01838-y)

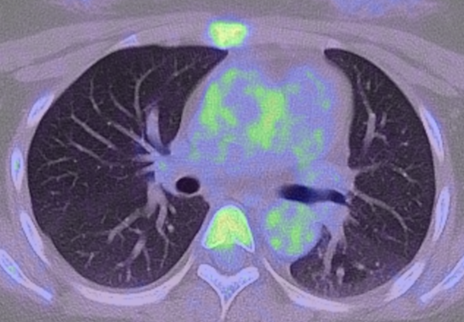

Supplement: Supplementary file 1 — Online Resource 1. PET-CT shows increased FDG accumulation in the aortic wall (white arrows). (DOCX 384 KB) [file 11748_2022_1838_MOESM1_ESM.docx]

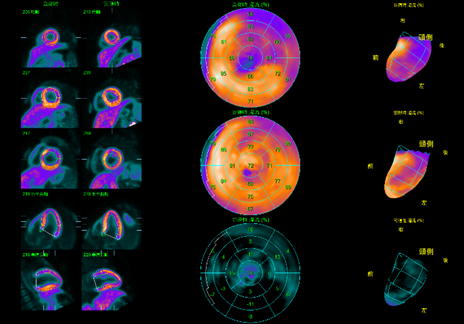

Supplement: Supplementary file 2 — Online Resource 2. N13 ammonia myocardial blood flow PET shows extensive ischemia. (DOCX 167 KB) [file 11748_2022_1838_MOESM2_ESM.docx]
